# Matrices, Their Uses and Retention

**Published:** 1903-08-15

**Authors:** Louis P. Hall


### MATRICES, THEIR USES AND RETENTION.

BY LOUIS P. HALL, D.D.S.

Read before the Michigan State Dental Association, July 9, 1903.

A matrix is a mold which gives form to material forced into it.

In dentistry it is used not only to give form to the filling, but by its use the cavity is changed from a difficult or complex form to a more simple one, thus facilitating the manipulation of the filling material, be it plastic or hard.

I am well aware that there are many who do not use a matrix at all. Some because they do not believe in them, some because *they* can do better without them, some because they haven't time, and some because they don't know how to use them.

The earlier and simpler forms of the matrix were made from pieces of gold plate, separating files, dress stays, etc. From time to time various improved matrices have been made and offered for sale. Of these each has its advantages and disadvantages. Matrices may be made from steel, German silver, or copper. The thickness, pliability or hardness of the material is much a matter of judgment and preference of the operator. In any case, as a rule, the thickness of material will depend upon the position of the teeth or space between them.

The pliability or stiffness of a matrix will depend much upon the form of the inter-proximal space and the method employed for holding the matrix to place, also the amount of force it will be required to withstand. With the latter improved forms of matrix retainers in which the support is evenly applied on both buccal and lingual sides and often from occlusal to gingival a much thinner and more pliable matrix can be used.

As the proximal cavities back of and including the cuspids present the most difficulties in filling, and especially the distal proximal cavities, it is in these that the matrix proves itself of most aid. If, however, it can be used on the mesial without obstructing either the view or the access, it will be of great help there.

With a matrix properly made and placed a filling can be inserted without so decided an undercut or form. A strong heavy wall may be had at the gingival, the necessity for the deep grooves or starting points are done away with, as the definite angle formed by the matrix and floor of cavity is usually sufficient for starting, and with a firm seat for the filling, anchorage may be had toward or on the occlusal surface.

If the matrix has been nicely adjusted, the filling at the cervical margin will require but very little, or at least much less, finishing then one done without a matrix. This alone is a great saving of time and vexation to both patient and operator, as it comes at the end of an already tiresome sitting.

Any matrix should be so shaped that when in position it will cut into or displace as little of the gum tissue as possible. If a thick matrix is used its edge should be beveled toward the tooth so that as it is pressed into place the plane surface will hug the surface of the tooth, and the beveled side will easily displace the free margin of the gum. All matrices, thick or thin, should be free from rough edges or points that would tend to irritate the gums or cut the dam, and should not be higher than the occlusal marginal ridge.

There are a few shapes and sizes of matrices that will fit a good many cases, but one should always have on hand materials of various kinds and thicknesses from which a matrix for a special case can be made.

If a case is presented in which not only the proximal wall is gone, but also more or less of either the buccal or lingual or some of each is lacking—and especially for heavy malleting—a heavy or stiff matrix can be used to best advantage. It would seem, though, that preference should be given to the thinner and more pliable materials when they can be held in position as well as form, for the reason that they are the more readily adapted to the shape of the tooth. The great objection to a stiff, springy matrix is that it is almost impossible to exactly fit it to the borders of the cavity, and unless it is so fitted when it is wedged up to place the spring tends to return to its original shape, instead of lying closely in place. Therefore, it would seem best to at least draw the temper from thick matrix material before shaping it.

If the cervical portion is held firmly up to border of cavity it does not matter so much if matrix at the side walls does give some. In fact, it is quite as well to allow a little space here to make sure of full contouring, as this portion of the filling is more readily trimmed.

In general, the cavity should be prepared in the usual way before the matrix is placed, and one should be especially careful to either disk or in some way dress off the proximal borders, so that the matrix will set smoothly around the tooth from that side.

One should be familiar with the shapes of the various teeth at the cervical border, remembering that the portion under the gum dips or slants toward the central axis of the tooth, so that even if it looks strong and broad it may be only a thin or frail edge of enamel.

Again, in the bucco-lingual direction the cervical marginal border of a cavity sometimes dips toward the central axis, that is, it is concave instead of straight, as in the proximal surfaces of the first upper bicuspid, where there many times exists a deep groove running rootwise; and if the matrix is is not fitted to that curve or a filling without a matrix is allowed to overlap at this curve, it is very difficult to remove the excess filling material, and if it is not removed it is always a source of annoyance and possibly a cause of recurrent caries.

The buccal and lingual proximal walls must be cut back if thin until they are thick enough to have considerable strength, and then the matrix should be closely drawn or pressed up to the sides of the cavity, especially to the cervical border.

In cases where the cavities are of so long standing that the teeth have fallen together from lack of proximal contact, the proximal walls must be cut back enough to allow for dressing them properly.

After the matrix is in place, separation of these teeth may be gained with much less danger to frail borders and walls, as the matrix will protect them.

I frequently use a matrix because it helps to carry the rubber down below the cavity margin when nothing else will do it. At other times I excavate all the rest of the cavity before touching the cervix wall, leaving that until the matrix is in place, because, by so doing, the matrix at that portion guides my instrument; thus I avoid disturbing the dam and gum and avoid bleeding and moisture at a part that is difficult to control.

How shall we hold a matrix? A piece of dry wood cut to wedge in two ways may be used. If coated with sandarac varnish it will hold better. But it has many disadvantages. If the space between the teeth is very narrow the wooden wedge must be made so thin that it is difficult to force it into place without breaking. Second, the "embrasure," as Dr. Black calls the spaces to the buccal and lingual of the contact point, being of unequal width and shape—a single wedge will not bring both sides up to place, unless the matrix be of stiff, heavy material. And it usually results in the loss of the proximal contact point, either because the matrix is too thick and there is too little separation or because the wedge is too near the contact point, in either case the matrix at the cervix is apt not to be tight. Then, too, the driving of a wedge or two wedges is very unpleasant to the patient, as it must all be done at once. If one of the various separators be used, even if the clamp had to be removed, both sides of the matrix can be brought up close to both the buccal and lingual borders of the cavity.

Until within the past two years the writer has almost always used some one of the separators for holding the matrix. Frequently holding two matrices, one to a mesial and another to a distal cavity at the same time.

One very great advantage of the separator for holding the matrix is that it is easily readjusted and after the first layer or two of material has been malleted to place—if more space be needed for contour work, or if the teeth begin to spread—a little tightening of the separator readjusts the matrix and supports the teeth.

When the fillings are completed, a little loosening of the separator permits the removal of matrix and you have the separator still in place for finishing the fillings especially on the proximal surface, and it is desirable that this should be done first so that the separator can be removed as soon as possible.

For the past year or so I have been using the Dickinson matrices and matrix retainer more or less. These matrices are of very thin steel and hence are quite pliable and easily drawn to place by the retainer. Their original shape is good, but I cut them a good deal for special cases. This retainer is made on the plan of the Elliot Ivory separator, excepting that in place of the two fixed points of the separator Dr. Dickinson has attached two double wedged-shaped points in such a manner that they readily adapt themselves to the shape of the slant of the embrasures, thus drawing the thin matrix up and giving it support along the entire lateral margins of the cavity.

Like almost all of our special instruments, this one will not work in all places. For instance, the cervical portions of the wedges do not always press in close enough to that border of matrix, and it has been the practice of some to insert a small piece of soft gutta-percha which, when hard, would cause the wedge to press up tight. It would seem however better to change the form of the wedge a little, and so I have now three sets of wedge points for the Dickinson retainer. They were about alike at first, but I have ground them in pairs—rights and lefts—with a little closer regard to the anatomy of the teeth. As the wedges are easily removed by a half turn of a little screw it is possible to combine any two of them and in that way meet almost any given case.

While with this retainer, as with the separator, the matrix can be easily readjusted and any amount of space for contour may be had, this retainer must be removed before the inter-proximal portion of a filling can be polished. It should be replaced with some other form of separator which will secure necessary space for finishing. This need not necessarily give much pain. as the teeth have already been separated.

Dr. William Crenshaw, of Atlanta, Ga., has recently given to the profession a set of matrices that seem in the hands of some to meet many requirements. These matrices and retainers are made upon the Perry separator plan. Instead of the separating points there are soft, steel duplex bands, which, as the tension bars are adjusted, may be drawn closely around the mesial and distal surfaces of the teeth giving the contour outline and at the same time providing separation for contour work. With this retainer it is often necessary to use a wooden wedge or a piece of gutta-percha to carry matrix close in at cervical margin.

The Crenshaw matrices and retainers are made in three sizes, for bicuspids, bicuspids and molars and for molars alone, though any one may be adapted to other places, but not readily.

Dr. Crenshaw has recently added a new form of matrix for anterior teeth consisting of a band of thin, flexible steel, which is stretched around the tooth having the cavity by a tension screw bar on the labial surfaces, thus making simple cavities.

Dr. G. W. Dittmar, in an able paper read before the Chicago Dental Society, suggests the following method of making and retaining a new form of matrix. I have not used it, but it would seem that it might be very effective in certain cases. He makes a matrix of 32 to 35 B. & S. gauge copper or German silver plate properly shaped to fit the case. The ends of these matrices he roughens by cutting in their ends where they lie against the buccal and lingual walls, five or six slits, not more than one half millimeter in depth for holding the ligature by which he retains the matrix in place. A small hole is made with a plate punch near the buccal and lingual corners next to the gingival edge through which the ligature is passed before adjusting the matrix, this will prove a great convenience in placing the matrix. By bending one or two flaps between the slits on both ends of the matrix the subsequent strands of ligatures will be prevented from slipping up or down. Having placed the matrix in position hold it so by tying the waxed silk ligature then encircle the tooth from three to six times, being careful that none of the threads are placed on the contact point. This method of adjusting the matrix is especially applicable when plastic materials are used for filling. If necessary for more support, a mechanical separator or wedge may be used.

Of the continuous band matrices on the market, the Brophy matrix has served a good purpose. The Lodge matrix appeals to me in many cases. The peculiar curve in which it is cut, together with its method of adjustment, enables one to bind the tooth very closely at the cervical margin while at the occlusal and it gives ample room for contour work. It is easily adjusted and does not unecessarily distend the cheek or lip.

It does not, however, provide separation in closely-set teeth, nor can you often so adapt it that both the mesial and distal parts of a very deep compound filling can be filled at the same time.

Another matrix which received more or less attention at the meetings last winter is designed for the restoration of crowns of teeth entirely with amalgam, and, too, by some to hold together without a band the roots of molars that have been separated either by decay or accident.

Pins are anchored in the roots. A band of soft steel or German silver about 30 gauge is passed around the exposed stump and the ends drawn tightly together with flat-nosed pliers, the band is then removed and soldered with easy-flowing solder as held in the pliers. It is again placed on the tooth or a stick and given proper contour and so trimmed that when placed in the mouth it does not interfere with occlusion. Into this is forced the alloy which is contoured above the band to suit the occlusion. The matrix may be left in the mouth until a subsequent sitting when it is cut and removed and the final contour and finishing done.

I think the only time I have had occasion to use a matrix in an anterior tooth was when the lingual marginal ridges of a superior lateral incisor failed to unite properly, and formed a groove which extended rootwise far beyond the gum margin. I had great difficulty in keeping the cavity dry until I hit upon the expedient of slipping a thin, curved matrix between the gum and the tooth and holding it in place with a tightly-drawn copper wire. With this device I was able to fill all under the gum with tin and finish the filling with gold.

An "S" shaped matrix is frequently of service in small proximal cavities that one does not feel warranted in cutting to the occlusal. Such cavities are sometimes found in the bicuspids and molars. A very little extension upon the side from which you elect to work will give access, and with a matrix drawn tight to the far side it is comparatively easy to fill.

By an "S" matrix I mean one that has a double or reverse curve from buccal to lingual, so that one point of a separator will draw the matrix against the tooth to be filled, while the other separator point will throw the other end of the matrix away from the portion of the cavity through which you fill, both points acting as usual for spaces.

I can not feel, as some do, that the matrix should be used only for plastic fillings. I believe that by the aid of the matrix many teeth can be successfully filled with gold that are usually filled with amalgam. And again, with the aid of a matrix many teeth could be filled with *something* which would otherwise be extracted if the patient could not afford a crown.

There are special objections to using a matrix on a mesial cavity. On the distal surface of a cavity the matrix border is in plain view, and it is comparatively easy to fill each point if care is used. On the mesial surface much care must be used, because the matrix tends to obscure the view and cut off the access, and unless great care is taken the operator need not be surprised when he has removed the matrix if he finds the borders unfilled. A mesial matrix for malleted work should be as narrow as possible and used perhaps only in starting a filling. Of course, no matrix should be used when there is free access and no closely-approximating teeth.

For plastic work a mesial matrix is as essential as is a distal for all cavities filled with amalgam must have continuous surrounding walls during the building of the filling.

It may not be out of place to say a word or two as to cavity preparation, especially at the margin next to matrix. The borders adjacent to the matrix should be cut at nearly right angles to the matrix, whether they are wholly on the proximal or to the buccal or lingual sides, so that acute angles which are difficult to fill may be avoided.

With the matrix in place the margins should again be examined carefully; sometimes, in drawing the matrix to place, the edges are liable to be chipped. This, of course, would make it difficult if not impracticable to make perfect adaptation of gold or other hard materials. Even amalgam would not be adapted as closely as it might be to a more perfect wall.

